# Cognitive Function in Genetic Generalized Epilepsies: Insights From Neuropsychology and Neuroimaging

**DOI:** 10.3389/fneur.2020.00144

**Published:** 2020-03-10

**Authors:** Corey Ratcliffe, Britta Wandschneider, Sallie Baxendale, Pamela Thompson, Matthias J. Koepp, Lorenzo Caciagli

**Affiliations:** ^1^Department of Clinical and Experimental Epilepsy, UCL Queen Square Institute of Neurology, London, United Kingdom; ^2^MRI Unit, Epilepsy Society, Chalfont St Peter, Buckinghamshire, United Kingdom; ^3^Department of Bioengineering, University of Pennsylvania, Philadelphia, PA, United States

**Keywords:** genetic generalized epilepsies, cognition, neuropsychology, neuroimaging, endophenotype

## Abstract

Genetic generalized epilepsies (GGE), previously called idiopathic generalized epilepsies, constitute about 20% of all epilepsies, and include childhood absence epilepsy, juvenile absence epilepsy, juvenile myoclonic epilepsy, and epilepsy with generalized tonic-clonic seizures alone (CAE, JAE, JME, and GGE-GTCS, respectively). GGE are characterized by high heritability, likely underlain by polygenetic mechanisms, which may relate to atypical neurodevelopmental trajectories. Age of onset ranges from pre-school years, for CAE, to early adulthood for GGE-GTCS. Traditionally, GGE have been considered benign, a belief contrary to evidence from neuropsychology studies conducted over the last two decades. In JME, deficits in executive and social functioning are common findings and relate to impaired frontal lobe function. Studies using neuropsychological measures and cognitive imaging paradigms provide evidence for hyperconnectivity between prefrontal and motor cortices, aberrant fronto-thalamo-cortical connectivity, and reduced fronto-cortical and subcortical gray matter volumes, which are associated with altered cognitive performance. Recent research has also identified associations between abnormal hippocampal morphometry and fronto-temporal activation during episodic memory. Longitudinal studies on individuals with newly diagnosed JME have observed cortical dysmaturation, which is paralleled by delayed cognitive development compared to the patients' peers. Comorbidities and cognitive deficits observed in other GGE subtypes, such as visuo-spatial and language deficits in both CAE and JAE, have also been correlated with atypical neurodevelopment. Although it remains unclear whether cognitive impairment profiles differ amongst GGE subtypes, effects may become more pronounced with disease duration, particularly in absence epilepsies. Finally, there is substantial evidence that patients with JME and their unaffected siblings share patterns of cognitive deficits, which is indicative of an underlying genetic etiology (endophenotype), independent of seizures and anti-epileptic medication.

## Introduction

Genetic generalized epilepsies (GGE) are a group of generalized epilepsy syndromes underpinned by high heritability and complex polygenetic inheritance (1, 2). Though GGE have traditionally been regarded as benign, recent research indicates specific profiles of cognitive impairment (3–5), particularly encompassing functions reliant on frontal lobe processing. Potential underlying mechanisms of cognitive dysfunction have been elucidated via advanced neuroimaging techniques, which allow quantifying morphological and functional brain changes as well as their relation to neuropsychological test scores.

The etiology of cognitive impairment in GGE is often regarded as neurodevelopmental (6, 7). Recent research has focused on profiling first-order relatives alongside index patients, in an effort to characterize the cognitive phenotypes of GGE subgroups while identifying familial traits with likely genetic underpinnings. General linear models, on the other hand, have been used to assess the relationship between cognitive impairment and disease-associated variables, including age at onset, duration of epilepsy, or the influence of specific anti-epileptic medication.

Relatively recent reviews have detailed the cognitive profiles of mixed GGE samples (4) or individual GGE syndromes, particularly JME (3), providing evidence of frontal lobe dysfunction. However, there is a scarcity of work focusing on potential syndrome-specific patterns of impairment, attempting to characterize the neural correlates of dyscognitive traits, or identifying potential determinants of such abnormalities. An updated view on these topics is therefore timely and compelling. Improved knowledge may aid clinical practice, by highlighting the extent of interventional needs, informing patient counseling, and identifying targets for cognitive rehabilitation and novel therapeutic approaches.

In this review, we will first summarize evidence on the overarching cognitive profile of GGE. We will then detail subsyndrome-specific investigations, which suggest slightly distinct patterns of dysfunction in juvenile myoclonic epilepsy and absence epilepsies. We will also elucidate recent structural and functional imaging research, which shed light on the putative abnormalities underlying cognitive dysfunction. Finally, we will discuss investigations assessing patients and their first-order relatives, which indicate genetic underpinnings as relevant determinants of cognitive profiles in GGE.

## Search Strategy and Selection Criteria

For this review, we conducted a literature search on PubMed ranging from January 1, 1985 to June 30, 2019, querying the following terms and related synonyms: “genetic generalized epilepsy,” “idiopathic generalized epilepsy,” “childhood absence epilepsy,” “juvenile absence epilepsy,” “absence epilepsy,” “juvenile myoclonic epilepsy”, in combination with the following individual key terms: “neuropsychology,” “neuropsychological,” “cognition,” “cognitive test,” “MRI,” “functional MRI/fMRI,” “family study,” “relatives,” “siblings,” “intermediate phenotype,” “endophenotype”. Searches were also repeated using common abbreviations of disease names (i.e., “IGE,” “GGE,” “CAE,” “JAE,” “JME”). We restricted our initial search to articles published in English. In addition, we carried out manual searches on reference lists of the identified articles and selected review papers published in the last 5 years, and complemented the former with extraction of relevant manuscripts from our records. Final inclusion was based on originality and direct relevance to the topics discussed in this Review.

## Cognitive Domains and Associated Neuropsychological Tests

The investigations reviewed in this manuscript implemented a variety of neuropsychological tests addressing different cognitive functions. Here, we briefly detail the most commonly assessed cognitive abilities and associated neuropsychological tests, to aid the interpretation of findings across studies. A more in-depth description of frequently used tests, parsed by cognitive domain, is provided in Table 1.

**Table 1:** Cognitive tests employed in GGE studies.

**Domain**	**Test (References)**	**Test description**
General intellectual ability	NART (8)	Requires the reading of 50 British English words with irregular spelling and unpredictable pronunciation
Processing Speed	Grooved Pegboard (9)	The participant is asked to place 25 pegs into 25 unique holes as quickly as possible (maximum time allowed: 3 min)
	Trail Making Test: Time—part A (10)	A series of numbers have to be connected in ascending order, using a continuous line, as quickly as possible
	(Digit–Symbol) Coding (WAIS) (11)	Visual symbols have to be assigned to an appropriate number, according to a given code pairing, as quickly as possible
	Stroop: Color–Word (12)	The maximum reading speed for color words and the naming speed of ink colors is recorded
Attention	Alertness tasks (13, 14)	The subject is asked to press a button instantaneously after viewing a stimulus, with and without a warning cue
	Vigilance task (13, 14)	The subject is asked to respond, as quickly as possible, to the omission of an expected switch of pattern between two squares. Testing lasts for 15 min
	Visual scanning task (13, 14)	The subject is asked to locate and react to a “critical stimulus” in a matrix of stimuli. The critical stimulus is not dissimilar enough from the other objects in the matrix so as to be obvious
	Posner Cueing task (15)	The subject is asked to respond to a stimulus, located to one side of a fixation point. A cue, which can either be congruent or incongruent, is used to “set” the directional attention of the participants, requiring an attentional shift in a proportion of the trials
Dexterity	Finger Tapping (16)	The participant is asked to tap the index finger on a lever as quickly as possible within a 10 s interval
Semantic knowledge	Vocabulary (WAIS) (11)	The participant is required to provide definitions for 33 unique words of increasing difficulty
	Similarities (WAIS) (11)	The subject is given 19 sets of word pairs and is asked to provide the common link (i.e., describe their relationship)
	Information (WAIS) (11)	The subject is asked a series of general knowledge questions of increasing difficulty
Visuo–spatial Abilities and Perceptual Reasoning	Block Design (WAIS) (11)	The participant is presented with a series of spatial problem-solving tasks of increasing difficulty, involving red and white cubes
	Matrix Reasoning (WAIS) (11)	The subject is required to complete a matrix of abstract patterns with one image missing
	ROCF—Copy (17, 18)	The participant is required to copy freehand a visually presented complex line drawing
Verbal generativity [Fluency can be considered an executive function reliant process, and is often included in executive function test batteries (21)]	Phonemic fluency—COWAT, “FAS Test” (10, 19, 20)	The subject is asked to generate as many words as possible starting with a given letter (F/A/S) in 1 min
	Semantic fluency—COWAT, “Animals, Fruit, and Vegetable Test” (10, 19, 20)	The subject is asked to generate as many category-specific words as possible (e.g., animals, fruits, vegetables) in 1 min
Expressive language (Naming)	McKenna Graded Naming test (22)	The participant is asked to name 30 items presented as black and white line drawings of graded difficulty.
	Boston Naming Test (23)	The participant is asked to name 60 items presented as black and white line drawings of graded difficulty.
	Auditory Naming (24)	The participant is asked to name 60 items based on verbal descriptions provided auditorily
Working memory	Digit Span (WAIS) (11)	The subject is required to repeat a set of numbers of increasing length in the correct order immediately upon presentation; this is followed by a second set in reverse order
	Spatial Span (WMS—III) (25) and Corsi Block Tapping test (26)	The participant is asked to copy block-tapping sequences of increasing length. Each trial, the number of taps required to complete a sequence increases by one
Verbal learning and memory	AMIPB: List learning (27)	The participant is required to learn a 15-item word list, presented auditorily over five trials, and recall that after a 15-item distracting list
	CVLT (28)	The participant is required to learn a 16-item word list over five trials and recall that after a 16-item distracting list, a long delay, and via a recognition task
	Logical memory I and II (WMS) (29)	The participant is required to recall an orally presented prose passage immediately (Condition I) and after a long delay (Condition II). A recognition task is incorporated in the delayed recall subtest
Non–verbal Learning and Memory	AMIPB: Design learning (27)	The subject is asked to reproduce a 9-element design on a 4 × 4 grid over five consecutive trials, and again following a distracting design
	ROCF—Recall (17, 18)	The subject is asked to copy a complex figure and then reproduce it from memory, shortly after presentation and after a 30 min delay' interval
	Designs I and II (WMS) (29)	The participant is presented with a series of unfamiliar designs. Short and long-term recall are measured by conditions I and II, respectively. The latter also probes visual recognition
Executive Functions	Stroop: Interference (12)	The subject is asked to name the ink color of color words written in incongruent color. Used as a measure of response inhibition.
	Trail Making Test: Task–switching (10)	The subject is asked to connect numbers and letters of the alphabet in sequence, alternating between letters and numbers, as quickly as possible. Used as a measure of cognitive flexibility.
	Five Points (30)	The subject is asked to create as many unique shapes as possible in 5 min, by connecting five symmetrical dots with straight lines. Used as a measure of strategy formation
	Tower of London (31)	The subject is asked to move colored shapes between three pegs in the minimum number of moves to achieve the required solution. Used as a measure of planning ability
	Wisconsin Card Sorting test (32)	Participants are asked to match cards in a stimulus set, but are not explicitly provided with rules. They are, however, told whether a match is correct. Cards are then sorted based on the implicit rules defined by the participant. The rules are then changed, and the participant is required to reformulate rules. Used as a measure of cognitive flexibility
	Hayling sentence completion (33)	The subject is asked to complete 15 sentences, each missing the last word, with an appropriate word. Subsequently, there are 15 sentences and the subject is required to provide a word that renders the sentence meaningless. Provides measures of response initiation and suppression, respectively
	Porteus Maze test (34)	The participant is asked to complete a set of variably complex mazes under time constraints. Used as a measure of planning ability
	Visual/Verbal test (35)	The subject is shown 42 cards, each depicting four objects, and asked to create a rule unifying three of the images on the card. They are then asked to create another one. Used as a measure of concept formation and cognitive flexibility
	Iowa Gambling task (36)	The participant is asked to win as much money as possible, by choosing from four decks of cards associated with variable gains and losses. Performance is dependent on reinforcement learning and identification of decks associated with advantageous choices. Used as a measure of decision making
	Ruff Figural Fluency Test (37)	The subject is asked to connect dots to create as many unique patterns as possible in 60 s. Used to measure strategy formation and non-verbal fluency

*AMIPB, Adult Memory and Information Processing Battery; COWAT, Controlled Oral Word Association Test; CVLT, California Verbal Learning Test; NART, National Adult Reading Test; ROCF, Rey–Osterrieth Complex Figure; WAIS, Wechsler Adult Intelligence Scale; WMS, Wechsler Memory Scale*.

General cognitive ability, often denoted by *g* or intelligence quotient (IQ), broadly refers to the ability of an individual to solve problems across multiple domains, independent of educational level (38). Full-scale IQ scores are formally derived after completion of a set of tests included in the Wechsler Intelligence Scale, currently in its fourth edition [WAIS-IV; (11)]. Abbreviated assessments, such as the National Adult Reading Test for British English speakers (8), are also available. The latter tests provide IQ estimates based on an individual's ability of reading words with irregular spelling, thus probing vocabulary, and produce scores that are highly correlated with IQ measures obtained via the Wechsler Scale.

Processing speed, defined as the maximum speed at which elementary cognitive operations can be executed (39, 40), involves efficient allocation of processing resources and tracking of ongoing tasks, and relies on intact attention and visuo-spatial skills. Frequently employed processing speed tasks include the Trail Making Test-A (10), requiring an individual to connect numbers in ascending order with a continuous line, or the Grooved Pegboard test (9), assessing an individual's ability to match pegs to unique holes. Attention, defined as the cognitive process enabling selective focus on specific stimuli while ignoring other perceivable information, is assessed via standardized test batteries (13, 14) quantifying levels of alertness, vigilance, visual scanning, cueing and ability to simultaneously concentrate on different tasks. While also relying on visuo-spatial abilities, intact attention represents a prerequisite for optimal executive control (41).

Other frequently administered tests, such as the Rey-Osterrieth Complex Figure test [ROCF; (17, 18)], include an initial “Copy” condition that entails an accurate reproduction of a visually presented complex line drawing, and thus assesses visuo-spatial constructional abilities. More generally, visuo-spatial processing is common to a multiplicity of cognitive domains, including perceptual reasoning, probed via WAIS subtests involving recognition of spatial relationships among items with increasing complexity (Block Design, Matrix Reasoning), and motor dexterity, which refers to fine motor skills and coordination. Tests addressing the latter rely on the correct execution of controlled sequential motor responses, such as those assessed via the Finger Tapping test (16).

With regards to language abilities, manipulation of acquired verbal information is often subsumed under the term of semantic knowledge, and is assessed via WAIS subtests including “Vocabulary,” “Similarities” and “Information,” which collectively probe general verbal knowledge attained through education and environmental exposure. Tests assessing auditory and visual confrontation naming, on the other hand, require naming items from their auditory description or from related black and white line drawings, respectively (22–24, 42). Verbal fluency, often categorized into phonemic and semantic fluency, refers to verbal generativity, and is probed via tests such as the Controlled Oral Word Association or “FAS” test, for the phonemic component, and animal naming for the semantic one (19, 20). These tasks require an individual to generate the largest possible number of words starting with a given letter, or to name as many items as possible belonging to a given category (i.e., animals, in most cases) in a specified time frame.

Working memory refers to the cognitive system responsible for the short-term storage of recently acquired information for manipulation and immediate use (43, 44), and is generally parsed into a verbal and a visuo-spatial component. Common working memory tests are represented by the Digit Span and Arithmetic tasks for verbal cues, included in the WAIS, and the Corsi Block-Tapping test (26), addressing visuo-spatial abilities. Tests such as the California Verbal Learning Test [CLVT; (28)], and the List Learning subtests of the Adult Memory and Information Processing Battery [AMIPB; (27)], assess the ability to encode and retain verbal cues, referred to as verbal learning and memory. Similar batteries are available for testing visuo-spatial learning and memory, such as the Design Learning subtest of the AMIPB or the recall phases of the ROCF, which require an individual to reproduce complex line drawings from memory. Similarly, measures of immediate and delayed verbal and visual learning and memory are also provided by the Wechsler Memory Scale [WMS; (29)].

A cognitive domain frequently included in the assessments of GGE, and closely related to information manipulation (45), is represented by executive functions, which encompass the high-order, top-down mental processes required to pay attention, concentrate, evaluate the efficacy of automatic responses and suppress “default,” stereotyped output when appropriate (41, 46). Response inhibition, concept formation, cognitive flexibility, goal selection, strategy usage, planning and monitoring are all examples of executive functions, and overall enable purposeful, self-serving and adaptive behavior. While language-based, verbal fluency tasks also require executive control, and are frequently included in test batteries addressing executive function (21). Traditionally, successful executive cognition relies on the integrity of the frontal lobes, particularly the prefrontal cortex, whose dorsolateral, ventrolateral and rostral subdivisions may exhibit some degree of functional specialization (47–50). There is a large variety of cognitive tests assessing dysexecutive traits, and the neuropsychological test batteries implemented by Wandschneider et al. (51), Moschetta and Valente (52), Jackson et al. (53), or Wandschneider et al. (54) may provide helpful examples.

## Genetic Generalized Epilepsies

GGE constitute about 20% of all epilepsies and are composed of four main subsyndromes: childhood absence epilepsy, juvenile absence epilepsy, juvenile myoclonic epilepsy, and epilepsy with generalized tonic-clonic seizures alone (CAE, JAE, JME, and GGE-GTCS respectively). Whilst varying with regard to age of onset, combination of different seizure types, EEG traits and disease courses, all GGE share a genetically determined multi-factorial etiology.

CAE, which presents with frequent typical absence seizures, has an age of onset which peaks at 6 to 8 years, an incidence of 0.7/100,000/year, and is twice as common in females as in males (55, 56). Age of onset for JAE peaks between 9 and 13. The syndrome is characterized by typical, though less frequent, absence seizures, often accompanied by generalized tonic clonic seizures (GTCS), and a similar distribution between males and females (57). Whilst CAE and JAE are two independent clinical entities, it is commonly surmised that these two disorders have highly overlapping etiology and pathological mechanisms. Consequently, the majority of investigations into their cognitive profiles have collapsed both diseases into the unitary category of absence epilepsy (AE). The hallmark of the most common GGE subsyndrome, JME, is represented by myoclonic jerks occurring in the morning. Most patients also suffer from GTCS and, more rarely, absence seizures. Disease onset peaks during adolescence and early adulthood, between 12 and 18 years of age (range: 5–25). JME likely represents 15–20% of all GGE cases, and is slightly more common in females (ratio of 3:2) (56, 58). Finally, GGE-GTCS has the most variable age of onset, generally ranging from the second to the fourth decade of life, and is believed to account for up to 15% of GGE, though prevalence estimates are often inconsistent (59).

## Cognitive Profiles of GGE: Neuropsychological Evidence

Although GGE have traditionally been regarded as benign, studies have consistently shown that executive functioning in GGE may be impaired. This has been complemented by research documenting a higher prevalence of impulsive personality traits (60, 61), cluster B personality disorders (62, 63), impaired emotion recognition and social cognition (64–66), suboptimal academic performance (53), and poor long-term social outcome (67, 68), particularly in JME. Moreover, meta-analytical syntheses of neuropsychological investigations conducted over the last three decades suggest that profiles of cognitive impairment may exhibit some degree of syndrome specificity (4). Here, we will discuss investigations of cognitive function in mixed groups of GGE patients, followed by studies detailing cognitive profiles in the most common GGE subsyndromes, juvenile myoclonic epilepsy and absence epilepsy.

### Cognition in Mixed GGE Samples

In mixed GGE samples, general cognitive ability is often reported as affected, with meta-analyses (4, 69) documenting IQ scores ranging from 0.5 to 1 standard deviation lower than controls, indicative of moderate to large effect sizes. However, whilst most investigations described lower general intelligence in GGE compared to controls, the IQ measures reported for GGE groups generally fall within the normal range, clustering around average values at the population level, i.e., between 90 and 110 (53, 70–73). Hence, it remains unclear whether general intellectual abilities in GGE may be lower than normative values, or whether differences between patient and control samples may arise, for instance, from the recruitment of high-performing, non-representative control cohorts across investigations.

Patients with GGE also exhibit reduced ability to manipulate acquired information, i.e., semantic knowledge. The recent meta-analysis by Loughman et al. (4) points to significantly lower scores in GGE compared to controls on tests such as the Vocabulary and Information items of the WAIS. In parallel, the latter meta-analytical synthesis also indicated impaired problem solving and reasoning abilities, elsewhere referred to as fluid intelligence. Two reports also documented poorer performance on standardized arithmetic tests, assessing both knowledge of mathematical operations and problem-solving skills, with scores of GGE patients up to one standard deviation lower than control subjects (53). Rathouz et al. (72) found that scores of arithmetic subtests were lower in GGE than in patients with focal epilepsy, and that both groups performed worse than healthy controls.

Across studies, there is homogenous reporting of worse dexterity, attention and processing speed in GGE, with all studies documenting moderate to substantial impairment in patients (53, 73–77). While evidence for disrupted motor and cognitive processing speed is consistent, and may point to altered visuo-motor integration, more research is required to address its potential determinants, particularly in regard to the detrimental influence of anti-epileptic medication. As several of these have been associated with cognitive slowing (78–80), the extent to which abnormal processing speed may thus represent an intrinsic feature of GGE, rather than a medication-associated effect, remains unestablished.

A smaller number of investigations indicate that phonological processing may also be impaired in GGE, with scores for letter and category fluency falling about one standard deviation below population-level normative ranges (60, 71, 74, 77). Jackson et al. (53) found that reading and measures of vocabulary did not differ between controls and patients with GGE, but reported a selective phonemic fluency deficit in the latter. More abundant evidence of abnormal verbal generativity, however, has been conveyed by investigations separately assessing individual GGE syndromes.

Evidence for working memory impairment is conflicting. Whilst some studies found significant deficits in mixed GGE groups compared to controls (60, 71, 74), other studies did not (53, 73, 81, 82). One investigation (74) detected differences between patients and controls for non-verbal attention performance, but no specific working memory dysfunction. Deficits in working memory are reported more often for the verbal (74) than for the non-verbal domain, suggesting greater compromise of the phonological loop than the visuo-spatial sketchpad, which refers to the subsidiary working memory construct accounting for visuo-spatial processing (44). Similarly, there is less concordant evidence for learning and memory impairment in GGE. While some authors suggest moderate to large effect sizes (74, 76, 82, 83), particularly for long-term memory in pediatric cohorts, other studies did not detect significant differences (81, 84), and confidence intervals of effect estimates appear fairly wide across all investigations (4). While these findings may point to syndromic heterogeneity, and warrant further consideration in the context of individual GGE syndromes, it overall appears that memory deficits may not be a specific GGE trait.

Finally, widely-documented impairment of both verbal and non-verbal fluency, strategy formation (73, 77), attention (53, 71), response inhibition (72), concept formation and mental flexibility (4) indicates moderate to pronounced executive dysfunction in GGE, pointing to abnormal frontal lobe function.

In summary, the available evidence in GGE conveys a cognitive profile characterized by average general intelligence along with consistent impairment of processing speed, dexterity, verbal generativity, and executive function. Literature supporting weak semantic knowledge, problem-solving and visuo-spatial reasoning is also available, though less abundant, whilst findings pertaining to working memory, learning and long-term memory performance are conflicting.

### Cognition in Patients With Juvenile Myoclonic Epilepsy

An overview of the studies assessing the cognitive profile of JME is provided in Table 2. General intellectual abilities are consistently found to be within the average range, though slightly lower than in controls (53, 73, 88–90, 94, 99). As discussed in section Cognition in Mixed GGE Samples, it is possible that differences in general intelligence between JME and controls may be partially ascribed to the investigation of high-performing control cohorts.

**Table 2:** Studies investigating cognitive function in JME.

**References**	**Design**	**Patients/Controls (n)**	**Patient Age (sd)**	**Age of Epilepsy Onset (sd)**	**Disease Duration (sd)**	**AED Regimen**	**Impaired Cognitive Domains**	**Unimpaired Cognitive Domains**	**Imaging**
Swartz et al. (85, 86)	C	9/15	28.0 (4.0)	9–20	N/A	Mixed	1. Working memory	1. Attention	FDG-PET
Devinsky et al. (87)	C	15/15	34.3 (N/A)	14.6 (N/A)	19.8 (N/A)	Mixed	1. Processing Speed^#^ 2. Abstract Reasoning^*^ 3. Executive Functions (Concept Formation^*^, Cognitive Flexibility^*^, Perseverative Tendencies^#^, Planning^#^)	1. Dexterity^#^	N/A
Sonmez et al. (88)	C	35/35	21.7 (4.5)	<25	7.2 (4.7)	Polytherapy	1. Visuo-spatial Perception (Cube Copying, Clock Drawing 2. Abstract Reasoning 3. Semantic Fluency 4. Verbal Learning and Memory 5. Non-verbal Learning and Memory 6. Executive Functions (Response Inhibition)	1. IQ 2. Visuo-spatial Perception (Facial recognition) 3. Expressive Language (Naming) 4. Working Memory	N/A
Kim et al. (89)	C	27/27	16–29	12–23	0.4–9	Drug-naïve	1. Processing Speed 2. Semantic Fluency 3. Working Memory 4. Verbal Learning 5. Executive Functions (Cognitive Flexibility)	1. General Cognitive Abilities 2. Verbal Memory 3. Non-verbal Memory	N/A
Pascalicchio et al. (90)	C	50/50	26.2 (7.4)	N/A	13.8 (8.5)	Monotherapy (VPA)	1. General Cognitive Abilities (IQ, VIQ, PIQ) 2. Processing Speed 3. Phonemic Fluency 4. Expressive Language (Naming) 5. Working Memory 6. Verbal Learning 7. Non-verbal Learning 8. Executive Functions (Cognitive Flexibility, Response Inhibition)	1. Semantic Knowledge (Information, Similarities) 2. Visuo-spatial Perception 3. Abstract Reasoning (Block Design)	N/A
Piazzini et al. (91)	C	50/40	37.3 (10.5)	19.0 (13.3)	18.3 (9.9)	Mixed	1. Phonemic Fluency 2. Executive Functions (Cognitive Flexibility)	1. General Cognitive Abilities (IQ)	N/A
Iqbal et al. (92)	C	8/16	28.1 (6.7)	N/A	N/A	Mixed	1. Phonemic Fluency 2. Semantic Fluency 3. Executive Functions (self-reported, questionnaire-based)	1. Processing Speed 2. Dexterity 3. Visuo-spatial Perception 4. Abstract Reasoning 5. Semantic Knowledge (Vocabulary) 6. Working Memory 7. Verbal Learning and Memory 8. Non-verbal Learning and Memory	N/A^$^
Roebling et al. (93)	C	19/20	24.2 (9.9)	N/A	N/A	Mixed	1. Phonemic Fluency 2. Semantic Fluency	1. Processing Speed 2. Attention 3. Semantic Knowledge (Vocabulary) 4. Working Memory 5. Verbal Learning and Memory 6. Non-verbal Learning 7. Executive Functions (Response Inhibition, Figural Fluency)	VBM and Working Memory fMRI
Wandschneider et al. (51)	C	19/42	25.5 (9.6)	N/A	11.1 (10.8)	Mixed	1. Attention 2. Semantic Knowledge (Vocabulary) 3. Semantic Fluency 4. Working Memory (Non-verbal) 5. Prospective Memory 6. Executive Functions (Response Inhibition)	1. Working Memory (Verbal) 2. Executive Functions (Cognitive Flexibility, Planning)	N/A
O'Muircheartaigh et al. (94)	C	28/55	33.6 (10.1)	14.4 (3.4)	20.2 (10.3)	Mixed	1. Semantic Knowledge (Similarities) 2. Phonemic Fluency 3. Expressive Language (Naming) 4. Non-verbal Learning 5. Cognitive Flexibility	1. General Cognitive Abilities 2. Semantic Knowledge (Vocabulary) 3. Semantic Fluency 4. Working Memory 5. Verbal Memory and Learning 6. Non-verbal Memory	VBM
Kim et al. (95)	C	25/30	25.3 (7.6)	14.7 (3.1)	10.6 (7.7)	Mixed	1. Processing Speed 2. Phonemic Fluency 3. Working Memory 4. Executive Functions (Cognitive Flexibility, Response Inhibition)	1. General Cognitive Abilities	Diffusion MRI
Moschetta and Valente (52)	C	42/42	26.6 (8.4)	14.0 (4.4)	17.8 (N/A)	Monotherapy (VPA)	1. Processing Speed 2. Phonemic Fluency 3. Working Memory 4. Executive Functions (Cognitive Flexibility, Response Inhibition)	N/A	N/A
Jackson et al. (53)	C	26/72	14.6 (3.1)	13.2 (4.1)	8.5 (3.5) (months)	96% Monotherapy	1. Processing Speed 2. Attention 3. Dexterity 4. Working Memory 5. Executive Functions (Problem Solving, Response Inhibition)	1. General Cognitive Abilities (VIQ, PIQ) 2. Semantic Knowledge (Vocabulary) 3. Phonemic Fluency 4. Expressive Language (Naming) 5. Verbal Learning and Memory 6. Executive Functions (Task-switching)	N/A
Lin et al. (96)	C	56/42	26.5 (9.0)	12.5 (4.6)	14.3 (10.0)	Mixed	N/A	1. General Cognitive Abilities 2. Vocabulary 3. Phonemic Fluency 4. Expressive Language (Naming) 5. Verbal Memory and Learning 6. Non-verbal Memory and Learning	MRI
Wandschneider et al. (97)	C	21/11	33.5 (22–64)^**^	N/A	N/A	Mixed	N/A	1. Processing Speed 2. Phonemic Fluency 3. Semantic Fluency 4. Working Memory 5. Executive Functions (Cognitive Flexibility, Decision Making^*^)	Working Memory fMRI
Zamarian et al. (98)	C	22/33	26.0 (18–50)^**^	14.0 (1–20)^**^	11.5 (3–45)^**^	Mixed	1. Processing Speed 1. Abstract Reasoning 2. Semantic Fluency 3. Executive Functions (Cognitive Flexibility, Planning, Decision Making)	1. Attention 2. Phonemic Fluency 3. Working Memory	N/A
Thomas et al. (99)	C	60^#^	31.0 (19–67)^**^	12.0 (8–15)^##^	21.0 (10–31)^##^	Mixed (Refractory to VPA)	1. General Cognitive Abilities (FSIQ, VIQ, PIQ) 2. Processing Speed 3. Semantic Knowledge (Vocabulary) 4. Abstract Reasoning (Block Design) 5. Phonemic Fluency 6. Semantic Fluency 7. Expressive Language (Naming) 8. Working Memory 9. Verbal Memory 10. Non-verbal Learning and Memory 11. Executive Functions (Response Inhibition)	1. Abstract Reasoning (Matrix Reasoning) 2. Verbal Learning 3. Executive Functions (Cognitive Flexibility)	N/A
Iqbal et al. (100)	C	22/44	26.7 (7.3)	N/A	N/A	Mixed	1. Dexterity (dominant hand) 2. Phonemic Fluency 3. Semantic Fluency	1. General Cognitive Abilities 2. Processing Speed 3. Dexterity (non-dominant hand) 4. Visuo-spatial Perception 5. Abstract Reasoning 6. Semantic Knowledge 7. Working Memory 8. Verbal Learning and Memory 9. Non-verbal Learning and Memory	N/A^$^
Giorgi et al. (101)	C	20/20	26.7 (6.6)	14.0 (3.8)	12.7 (8.4)	Mixed	1. Processing Speed 2. Semantic Fluency 3. Working Memory 4. Verbal Learning 5. Non-verbal Learning and Memory	1. Phonemic Fluency 2. Verbal Memory 3. Executive Functions (Cognitive Flexibility, Response Inhibition)	N/A
Valente et al. (102)	C	57/44	27.4 (8.2)	N/A	N/A	Monotherapy (VPA)	1. Processing Speed 2. Phonemic Fluency 3. Working Memory 4. Verbal Memory and Learning 5. Non-verbal Memory and Learning 6. Executive Functions (Cognitive Flexibility, Response Inhibition)	N/A	N/A
Abarrategui et al. (73)	C	19/21	33.0 (8.1)	14.0 (12–16)^**^	18.0 (14–25)^**^	Mixed	1. Processing Speed	1. General Cognitive Abilities 2. Semantic Knowledge (Information) 3. Visuo-spatial Perception/ Orientation 4. Abstract Reasoning 5. Phonemic Fluency 6. Expressive Language (Naming) 7. Working Memory 8. Verbal Memory 9. Non-verbal Memory 10. Executive Functions (Cognitive Flexibility, Response Inhibition, Planning)	N/A^$^
Rzezak et al. (103)	C	79/69	27.3 (8.4)	N/A	N/A	Mixed	1. Processing Speed 2. Phonemic Fluency 3. Semantic Fluency 4. Working Memory 5. Executive Functions (Cognitive Flexibility, Response Inhibition)	N/A	
Sezikli et al. (104)	C	45/15	22.9 (6.8)	15.6 (4.1)	7.2 (5.6)	Monotherapy (VPA)	1. Processing Speed (Trail Making A) 2. Semantic Fluency 3. Working Memory 4. Non-verbal Memory 5. Executive Functions (Cognitive Flexibility, Figural Fluency)	1. Processing Speed (Stroop CW) 2. Attention 3. Verbal Memory 4. Executive Functions (Response Inhibition)	N/A
Unterberger et al. (105)	C	36/38	25.3 (5.3)	14.3 (3.4)	N/A	Mixed	1. Processing Speed 2. Attention 3. Executive Functions (Risk taking)	1. General Cognitive Abilities (VIQ) 2. Phonemic Fluency 3. Semantic Fluency 4. Executive Functions (Cognitive Flexibility, Response Inhibition)	N/A
Paiva et al. (106)	C	35/39	29.0 (9.1)	15.7 (5.2)	13.7 (9.4)	Mixed	1. Executive Functions (Risk taking)	1. Executive Functions (Decision Making under ambiguity)	N/A

*Studies are listed in chronological order. Unless specified otherwise, age, age of epilepsy onset and disease duration are reported as mean values in years, or as ranges, if provided in such format by the original reference*.

** *Median (range)*.

## *Median (interquartile range)*.

$ *Studies employing video-EEG during neuropsychological testing. C, Cross-sectional design; CW, Color-Word (Stroop test); IQ, Intelligence Quotient; PIQ, Performance Intelligence Quotient; VBM, Voxel-Based Morphometry; VIQ, Verbal Intelligence Quotient; VPA, Sodium Valproate*.

In Devinsky et al. (87): ^*^, reduced function in JME compared to TLE; ^#^, comparisons against healthy controls. In Wandschneider et al. (97): ^*^, shift toward more advantageous choices (i.e., task-associated learning) was impaired in JME patients with ongoing seizures, but not in those who were seizure-free. In the “AED Status” column, “Mixed” is given for studies where AED use was not restricted to a single regimen (i.e., monotherapy, polytherapy, or drug-naïve).

Across studies summarized in the meta-analysis by Loughman et al. (4), there is evidence for consistent impairment of semantic knowledge and problem-solving skills, which recapitulates findings in mixed GGE samples. With regards to visuo-spatial abilities, visual attention has also been reported as impaired in JME (89, 90, 100, 104). While a meta-analytical synthesis (4) and more recent evidence (73, 104) suggested, on the other hand, that visuo-spatial thinking may be relatively intact, other findings (88) implicated minor visuo-spatial dysfunction, as assessed via clock drawing and cube copying tests. In line with evidence in mixed GGE samples, a number of studies documented impaired dexterity and processing speed (53, 73, 87, 90, 99, 100, 104, 105), with patients often performing more than one standard deviation below controls.

In relation to phonological processing, impairment of phonemic and semantic fluency was detailed in early investigations (88, 90) and confirmed by a large number of subsequent studies. Performance levels ranging between 0.5 and 1 standard deviation lower than controls have been reported by most investigations, indicative of moderate to consistent dysfunction (51, 91–95, 99, 100, 104). Medication-related effects might be involved, but have not yet been specifically addressed. Moschetta and Valente (52), for instance, highlighted an association between sodium valproate usage and worse performance on several cognitive tasks, including those assessing verbal fluency. As patients taking higher doses of valproate had a higher seizure frequency, however, it remains unclear whether worse executive performance may relate to epilepsy severity, anti-epileptic medication, or both the former. Information regarding treatment with topiramate, a drug commonly associated with adverse cognitive effects (107), was also lacking in several of the above investigations.

Most studies into working memory in JME reported some degree of impairment (90, 95, 99, 101). Other groups have examined dimension-specific performance, with some finding evidence for visuospatial impairment (51, 85, 86, 94, 101), and others documenting deficits in verbal working memory (52, 89, 101). While only few reports documented normal functioning (93, 100), whether working memory weaknesses may be more prominent in the verbal than non-verbal domain remains unclear.

Dysexecutive traits are very commonly described for JME, and may represent its hallmark. The typical profile encompasses impairment of response inhibition (51, 53, 90, 95, 99, 102), attention, goal maintenance, concept building, problem solving, task-switching, and cognitive flexibility (52, 53, 87, 89, 91, 94, 104). Two studies attempted within-groups stratification of effects, documenting moderate to severe deficits in executive functions in 83% and 68% of the respective samples (52, 99). Of note, however, Thomas et al. (99) explicitly focused on difficult-to-treat patients with JME, who had not experienced seizure freedom with sodium valproate. It is also reported that JME patients may experience more “everyday life problems” as a result of dysexecutive traits (92, 100). Decision-making, another high-level executive function, also appears affected. Patients with JME may exhibit difficulties in making advantageous decisions under ambiguity (98), and commit to more risky choices than controls (105, 106). Interestingly, Wandschneider et al. (97) suggested that risky decision making may be particularly relevant in the patient subgroup with poorly controlled seizures, pointing toward an interplay between epilepsy severity and cognitive outcome.

Prospective memory, a system of creating, retaining, and implementing prior intentions and plans, is heavily reliant on executive functions, and has been evaluated via a complex multi-step task (51). At the intention formation stage, patients with JME developed more rudimentary plans than controls, suggesting impaired planning and cognitive flexibility. Furthermore, patients also completed significantly fewer tasks, suggestive of deficits in the executive component underlying prospective memory.

The involvement of cognitive functions reliant on temporal and hippocampal processes in the JME profile is uncertain. Several studies reported normal levels of functioning on tests of learning and memory (51, 53, 73, 93, 96, 100, 108), whereas others detailed deficits in short and long-term recall when compared to controls (4, 90, 99, 101). Impaired memory has been considered a consequence of impoverished visual and verbal learning (88, 89, 104). Conflicting evidence may be partially due to syndromic heterogeneity.

Some reports have suggested that heterogeneity of cognitive performance in JME may relate to compensatory strategies, dependent on general intelligence level (103). While it can be argued that higher IQ in a proportion of JME cases may relate to more effective strategy formation, enabling successful compensation and thus normative executive performance, the hypothesis of IQ as a protective factor for cognitive dysfunction in JME lacks strong empirical support. Moschetta et al. (52) previously reported that cognitive performance in most domains was lower in JME than controls even after co-varying for IQ, thus suggesting independence of effects.

On balance, studies investigating cognition in JME documented average general intelligence, which is however paralleled by impairment of verbal generativity, working memory and a wide range of executive functions, with moderate to large effect sizes. Semantic knowledge, reasoning, processing speed and dexterity also appear affected, while evidence regarding learning and memory deficits is conflicting. Finally, the literature is overall not supportive of impairment of visuo-spatial abilities.

### Cognition in Patients With Absence Epilepsies

Table 3 summarizes findings of the investigations assessing cognitive function in CAE and JAE, often subsumed under the unitary category of AE, as specified earlier. Seminal research from Pavone et al. (109) found that AE may present with a subtle lowering of IQ compared to controls, which is corroborated by a recent review and several investigations (5, 73, 113, 114). As for mixed GGE samples and JME, however, IQ values are generally reported as within average ranges for the majority of AE patients. It is suggested that general cognitive ability may negatively correlate with disease duration (110, 111).

**Table 3:** Studies investigating cognitive function in AE.

**References**	**Design**	**Patients/Controls (n)**	**Patient Age (sd)**	**Age of Epilepsy Onset (sd)**	**Disease Duration (sd)**	**AED Regimen**	**Impaired Cognitive Domains**	**Unimpaired Cognitive Domains**	**Imaging**
Pavone et al. (109)	C	16/16	9.2 (3.0)	5.3 (3–8)^**^	N/A	Mixed	1. General Cognitive Abilities (IQ) 2. Visuo-spatial Skills 3. Non-verbal Learning and Memory	1. Semantic Knowledge 2. Verbal Memory	N/A
Henkin et al. (74)	C	12/20	14.4 (1.83)	7.2 (4–11)^*^	N/A	Monotherapy (VPA)	1. Attention 2. Semantic Fluency 3. Verbal Learning and Memory	1. Dexterity 2. Phonemic Fluency 3. Non-verbal Learning and Memory	N/A
Caplan et al. (110)	C	69/103	9.6 (2.5)	6.2 (2.5)	3.5 (2.8)	Mixed	1. General Cognitive Abilities (FSIQ, PIQ, VIQ) 2. Spoken Language Quotient	N/A	N/A
Caplan et al. (111)	C	78/102	N/A	N/A	N/A	Mixed	Same as above	Same as above	N/A
Conant et al. (112)	C	16/29	8.0 (1.3)	4–8	13.8 (8.5)	Mixed	1. Dexterity 2. Attention 3. Phonemic Fluency 4. Executive Functions (Cognitive Flexibility, Planning and Integration)	1. Processing Speed 2. Semantic Fluency 3. Verbal Memory 4. Non-verbal Memory 5. Executive Functions (Response Inhibition)	N/A
Vega et al. (113)	C	38/46	10.5 (2.3)	6.9 (2.8)	3.4 (2.7)	Mixed	1. Attention	N/A	N/A
Tosun et al. (114)	C	24/28	9.2 (2.2)	7.0 (2.0)	2.3 (2.2)	Mixed	1.General Cognitive Abilities (FSIQ, VIQ)	1.General Cognitive Abilities (PIQ)	SBM
D'Agati et al. (115)	C	15/15	11.4 (2.2)	8.8 (1.7)	2.7 (1.3)	Monotherapy (VPA)	1. Processing Speed 2. Phonemic Fluency 3. Semantic Fluency 4. Executive Functions (Task-switching)	1. Working Memory 2. Verbal Memory 3. Non-verbal Memory 4. Executive Functions (Planning)	N/A
Kernan et al. (116)	C	31/51	9.0 (2.0)	6.0 (2.0)	3.0 (2.0)	Mixed	1. Verbal Learning and Memory (CLVT and Stories) 2. Executive Functions (Cognitive Flexibility)	1. General Cognitive Abilities 2. Processing Speed 3. Working Memory 4. Verbal Memory and Learning (Doors and People) 5. Non-verbal Learning and Memory 6. Executive Functions (Response Inhibition)	N/A
Jackson et al. (53)	C	11/72	12.2 (3.5)	11.2 (3.5)	9.7 (3.2) (months)	Mixed	1. General Cognitive Abilities (VIQ, PIQ, Spelling) 2. Attention 3. Dexterity 4. Phonemic Fluency 5. Expressive Language (Naming) 6. Working Memory 7. Executive Functions (Problem Solving, Response Inhibition)	1. Processing Speed 2. Semantic Knowledge (Vocabulary and Reading) 3. Verbal Learning and Memory 4. Executive Functions (Task-switching)	N/A
Masur et al. (117)	L	446/N/A	N/A	N/A	N/A	Mixed	1. Attention	1. General Cognitive Abilities 2. Processing Speed 3. Semantic Knowledge (Vocabulary) 4. Working Memory 5. Verbal Memory 6. Non-verbal Memory 7. Executive Functions (Cognitive Flexibility)	N/A
Cheng et al. (118)	C	37/37	8.0 (2.3)	6.2 (1.5)	N/A	Mixed	1. General Cognitive Abilities 2. Attention 3. Processing Speed 4. Executive Functions (Cognitive Flexibility)	1. Visuo-spatial Perception 2. Working Memory 3. Verbal Learning and Memory 4. Non-verbal Learning and Memory	N/A
Cheng et al. (119)	C	35/33	7.3 (1.3)	6.7 (1.3)	7.0 (7.0) (months)	Drug-naïve	1. General Cognitive Abilities 2. Attention 3. Executive Functions (Cognitive Flexibility, Problem Solving)	1. Processing Speed 2. Visuo-spatial Perception 3. Semantic Knowledge 4. Working Memory	N/A

*Studies are listed in chronological order. Unless specified otherwise, age, age of epilepsy onset and disease duration are reported as mean values in years, or as ranges, if provided in such format by the original reference*.

* *Mean (Range)*;

** *Median (Range). C, Cross-sectional design; CVLT, California Verbal Learning Test; FSIQ, Full-Scale Intelligence Quotient; IQ, Intelligence Quotient; L, Longitudinal design; PIQ, Performance Intelligence Quotient; SBM, Surface-Based Morphometry; VIQ, Verbal Intelligence Quotient; VPA, Sodium Valproate. In the “AED Status” column, “Mixed” is given for studies where AED use was not restricted to a single regimen (i.e., monotherapy, polytherapy, or drug-naïve). Kadish et al. (120) outlined a validation of a screening tool for attention and executive function, but did not provide individual subtest scores, hence could not be included in the table*.

Phonological processing represents one of the most frequently described domains of cognitive impairment in AE (5), and relates to reduced linguistic abilities, semantic knowledge, verbal intelligence quotient [VIQ; (53)] and spoken language quotient [SLQ; (110)]. Decline in several aspects of linguistic functioning may be associated with disease duration (111). Alongside expressive naming (53, 74, 116), both semantic and phonemic fluency have been found as weak in AE, with performances falling one standard deviation below those of normative controls (112, 115).

Early reports also documented impoverished performance on tests of visuo-spatial skills in AE, as measured by the Performance IQ (PIQ) component of the WISC-R (53, 109). This was associated with relatively poor scores on tests of dexterity (53, 74, 112) and processing speed (112, 115). Abstract visuo-spatial reasoning and line orientation may also be poorer in AE patients than controls (73). Most research has not found evidence for working memory deficits in AE, though a recent study suggest impairment of its visuo-spatial component (73). It is possible that this finding may be a consequence of more general disruptions in visuo-spatial processing.

As a distinguishing feature of AE, several studies reported impairment of attentional control, affecting both verbal and non-verbal modalities (73, 74, 112, 115–118, 120). In the largest investigation to date, involving over 400 individuals with newly diagnosed, drug-naïve CAE, attentional deficits were reported in more than a third of probands despite average intelligence, and persisted 16–20 weeks after treatment initiation, even when successful seizure control was attained (117). Moreover, causal modeling indicated downstream sequential effects of attentional deficits on memory, executive function and academic achievement (117), corroborating early reports that proposed impaired attention as the underlying mechanism for poor memory performance (109). Reduced attentional skills were elsewhere found associated with higher levels of distractibility and forgetfulness (113) and lower arithmetic proficiency (119).

Though impaired attention is the predominant finding in AE, dysexecutive traits are also reported in AE samples, in accord with typical findings in GGE, and include reduced scores for measures of problem-solving, response inhibition, processing speed, planning and mental flexibility (53, 112, 115, 118). Jackson et al. (53) indicated that impairment of attention and executive skills is clinically relevant, with performance of more than one standard deviation below normative levels in patients. We did not identify any investigation exploring decision-making or prospective memory in AE patients.

As in JME, evidence for impaired functions relying on mesiotemporal involvement in AE is controversial (5). Pavone et al. (109) reported abnormal non-verbal learning and memory, along with impaired delayed recall. Impoverished performance on standardized spelling tests has also been suggested as a potential indicator of altered long-term memory (53, 74, 116). Other studies, however, have found comparable performance on tests of learning and memory in patients and controls (73, 112, 115, 121). It is possible that learning and memory deficits may not be specific, and arise as a consequence of impaired phonological processing.

Lower IQ and impaired phonological ability in AE may be associated with anti-epileptic medication usage and disease duration. In the largest randomized controlled trial to date, sodium valproate appeared associated with significantly more frequent attentional deficits than ethosuximide and lamotrigine, independent of treatment response (117). Reduced FSIQ and PIQ appeared more prominent at a younger age and/or earlier age at disease onset than linguistic deficits, indicating a possible neurodevelopmental mechanism and differential modulatory effects of disease-related-variables (111). In a study considering cognitive dysfunction independently across GGE subsyndromes, Abarrategui et al. (73) posited that AE may present with the most severe cognitive impairment of all GGE, based on the assessment of a medicated cohort with a long disease duration (mean = 24.5 years). Other studies, however, report smaller effect sizes. On balance, it is maintained that inadequate seizure management relates to poor cognitive prognosis (68).

On balance, neuropsychological investigations in absence epilepsies also indicate average general intelligence, but principally substantiate impairment in two domains: phonological processing, which relates to most stages of language production and semantic knowledge, and attention, which represents the most commonly affected skill, and may in turn detrimentally affect executive function. Contrary to evidence in JME, however, there is a relative paucity of reports addressing high-level dysexecutive traits, and no evidence of altered decision making or risk-taking behavior. It remains to be established whether the latter traits may be specific to JME. Finally, while evidence for impaired verbal generativity is also widely documented for JME, its presence is mostly emphasized within the broader context of dysexecutive traits, rather than globally dysfunctional linguistic abilities. Future analyses directly comparing JME and AE across a test battery addressing language performance may shed further light on potential syndrome-specific cognitive features.

## Neural Correlates of Cognitive Impairment in GGE

By ILAE definition, patients with GGE present with normal clinical MRI. Advanced post-processing methods such as voxel-based morphometry (122), surface-based MRI analysis (123), diffusion tensor imaging [DTI; (124)], and functional MRI have identified widespread structural and functional abnormalities in GGE, mostly implicating fronto-cortico-thalamic regions and their connections (125–133).

During the generalized spike-wave paroxysms typical of GGE, combined EEG-fMRI studies have documented the involvement of the thalamus and fronto-parietal cortices, mostly overlapping with default-mode network (DMN) areas (134–137). Overall, these findings have led to the conceptualization of GGE as disorders of thalamo-cortical connectivity. The diffuse abnormalities of cortical and subcortical structure, function, and connectivity in GGE may also relate to altered cognitive functioning, and most studies have investigated the neural correlates of cognitive function in separate GGE subsyndromes. Findings are summarized in Table 4.

**Table 4:** Studies investigating imaging correlates of cognitive function in JME and AE.

**Reference**	**Design**	**Patients/Controls (n)**	**Patient Age (sd)**	**Age of epilepsy onset (sd)**	**Disease duration (sd)**	**AED regimen**	**Summary**
**JME**							
Swartz et al. (86)	C	9/14	28.0 (4.0)	9–20	N/A	Polytherapy	FDG-PET− 1) Rest: ventral premotor, dorsolateral frontal, temporal, limbic and caudate hypometabolism in JME 2) Working Memory: dorsolateral frontal, premotor and basal frontal hypometabolism, fusiform and temporo-polar hypermetabolism
Savic et al. (138)	C	26/10	30.6 (7.7)	13.6 (3.0)	17.2 (8.2)	Mixed	MR Spectroscopy—Reduced processing speed and cognitive flexibility scores in JME patients with lower frontal lobe N-Acetyl Aspartate concentration
McDonald et al. (139)	C	10/14	27.9 (4.7)	N/A	N/A	N/A	FDG-PET—No frontal hypometabolism in JME. Bilateral orbito-frontal and premotor metabolism related to non-verbal fluency, bilateral frontal hypometabolism associated with mental flexibility
Pulsipher et al. (140)	C	20/51	15.5 (2.8)	14.5 (3.0)	8.9 (3.7) (months)	Mixed	Structural MRI—Smaller thalamic volumes and increased frontal cerebrospinal fluid in JME. Thalamic volumes related to cognitive flexibility in the JME and control groups, frontal gray matter associated with cognitive flexibility and response inhibition in the JME group only
Roebling et al. (93)	C	19/20	24.2 (9.9)	N/A	N/A	Mixed	Structural MRI and working memory fMRI—No gray matter volume differences between patients with JME and controls, and no intergroup activation differences during a verbal and a visuo-spatial working memory task
O'Muircheartaigh et al. (94)	C	28/55	33.6 (10.1)	14.4 (3.4)	20.2 (10.3)	Mixed	Structural MRI—in JME, fractional anisotropy of anterior SMA positively correlated with naming performance, fractional anisotropy and gray matter volume of the posterior cingulate cortex negatively correlated with processing speed
Vollmar et al. (141)	C	30/26	32.8 (9.9)	N/A	N/A	Mixed	Working memory fMRI−1) abnormal co-activation of motor cortex and SMA with high cognitive load, and 2) impaired deactivation of the default-mode network in JME
Kim et al. (95)	C	25/30	25.3 (7.6)	14.7 (3.1)	10.6 (7.7)	Mixed	DTI—Impairment of processing speed, phonemic fluency, working memory, cognitive flexibility, and response inhibition in JME not correlated with fractional anisotropy or mean diffusivity abnormalities
O'Muircheartaigh et al. (129)	C	28/27	34.1 (9.9)	14.8 (2.7)	8.7 (11.5)	Mixed	Language fMRI—Phonemic fluency scores associated with attenuation of thalamocortical connectivity during verbal fluency paradigm, which was defective in JME
Lin et al. (96)	C	56/42	26.5 (9.0)	12.5 (4.6)	14.3 (10.0)	Mixed	Structural MRI—In JME, hippocampal volumes associated with performance on tests of semantic knowledge, phonemic fluency, verbal memory and learning
Wandschneider et al. (97)	C	21/11	33.5 (22–64)^**^	N/A	N/A	Mixed	fMRI—Poor decision-making associated with bilateral dorsolateral frontal activation in JME, and with reduced DMN deactivation in controls. Performance in JME patients with ongoing seizures negatively correlated with dorsolateral frontal activation. Non-learners had stronger activation of pre-SMA, left dorsolateral frontal cortex, and right superior frontal gyrus than learners
Lin et al. (142)	L^#^	19/57	14.9 (0.7)	14.0 (0.7)	8.4 (0.9)	Mixed	MRI—Lower longitudinal improvement in IQ, processing speed, and response inhibition scores in JME related to attenuation of the expected cortical thinning and surface area reduction in fronto-temporo-parietal association areas
Caeyenberghs et al. (143)	C	35/35	26.8 (7.8)	15.0 (3.5)	15.2 (8.8)	Mixed	Structural MRI—Tractography-based connectivity between right precuneus and left postcentral gyrus positively correlated with VIQ, naming, abstract reasoning, and verbal memory. Connectivity between right hippocampus and right postcentral gyrus also associated with abstract reasoning.
Caciagli et al. (108)	C	37/36	32.0 (14.0)^***^	15.0 (4.0)^***^	19.0 (16.0)^***^	Mixed	Structural MRI—IQ and memory scores not associated with hippocampal malrotation in JME. Memory fMRI—Abnormal mesiotemporal and dorsolateral frontal activation in all JME patients during verbal memory, reorganized mesiotemporal activation for visual memory in JME with hippocampal malrotation only
**AE**							
Caplan et al. (144)	C	26/37	9.7 (2.1)	6.9 (2.1)	2.2 (2.3)	Mixed	Structural MRI—Gray matter volume loss in left orbital frontal gyrus and bilateral temporal lobes in CAE. Volume of these areas related to IQ in controls, not in patients.
Killory et al. (145)	C	26/22	12.0 (4.0)	N/A	N/A	Mixed	EEG-fMRI—Decreased medial frontal fMRI activation associated with poorer continuous performance test results in CAE. Concomitant impaired connectivity within attentional networks in CAE compared to controls
Tosun et al. (114)	C	24/28	9.2 (2.2)	7.0 (2.0)	2.3 (2.2)	Mixed	Structural MRI (SBM) 1) *Sulcal depth:* PIQ and VIQ less associated with medial/superior frontal, superior temporal, and occipito-parietal sulcal depth in CAE than controls, and more associated with middle frontal sulcal depth in CAE than controls 2) *Cortical thickness:* frontal and temporal thickness less associated with PIQ and VIQ in CAE than controls, while orbito-frontal thickness is more associated with PIQ and VIQ in CAE
Lin et al. (146)	C	21/27	9.6 (2.1)	7.0 (2.1)	2.6 (2.5)	Mixed	Structural MRI—in CAE, no association between thalamic volumes and cognitive measures (IQ, SLQ), but negative correlation detected between left thalamic volume and scores on a social problem assessment scale.
Guo et al. (147)	C	39/ no controls	9.9 (3.1)	N/A	3.0 (2.5)	Medication withheld 48h prior to scanning	EEG-fMRI during tasks—Absence seizures with behavioral impairment during finger tapping and attention tasks associated with more marked fMRI signal increases in default-mode, fronto-parietal and thalamic-/sensory-motor network than seizures with no impairment in task performance.

*Studies are listed in chronological order. Unless specified otherwise, age, age of epilepsy onset and disease duration are reported as mean values in years, or as ranges, if provided in such format by the original reference*.

** *Median (range)*.

*** *Median (interquartile range)*.

# *Demographics are provided for the sample at baseline. C, Cross-sectional design; CAE, Childhood Absence Epilepsy; DMN, Default Mode Network; DTI, Diffusion Tensor Imaging; (f)MRI, (Functional) Magnetic Resonance Imaging; IQ, Intelligence Quotient; JME, Juvenile Myoclonic Epilepsy; L, Longitudinal Design; PIQ, Performance Intelligence Quotient; SBM, Surface Based Morphometry; SLQ, Spoken Language Quotient; SMA, Supplementary Motor Area; VIQ, Verbal Intelligence Quotient. Mixed AED status is given for studies where AED use was not restricted to a single regimen (i.e., monotherapy, polytherapy, or drug-naïve)*.

### Neural Correlates of Cognitive Impairment in JME

In JME, early functional imaging studies aimed to identify the neural correlates of working memory and executive dysfunction. The first positron emission tomography (PET) investigation documented an association between impaired working memory performance in JME and reduced 18-fluorodeoxyglucose uptake within premotor, anterior frontal cortices and caudate nucleus (86). Subsequently, McDonald and collaborators detected an association between frontal PET hypometabolism and lower mental flexibility scores (139). In an MR-spectroscopy study, Savic and colleagues reported reduced frontal lobe N-Acetyl Aspartate (NAA) concentrations, a marker of neuronal damage or dysfunction, in JME patients (148). Low frontal NAA was more prominent in those with poorer performance on an abbreviated cognitive assessment addressing frontal lobe function (138). Collectively, these early investigations provided complementary evidence linking dysexecutive traits to markers of impaired frontal lobe function across imaging modalities.

Subsequent investigations assessed the neural underpinnings of cognitive function in JME using task-based fMRI. Initial reports did not detect activation differences between JME patients and controls during a working memory fMRI task, which included verbal and visuo-spatial modified versions of the Sternberg Item Recognition Test (93). More recently, however, Vollmar and collaborators identified abnormal motor co-activation and increased functional connectivity between motor system and prefrontal cognitive networks during a visuo-spatial working memory task, which entailed joystick usage (141). While not substantiating the pattern of “hypofrontality” suggested by early imaging work, these findings point instead to an altered interplay between functionally segregated brain networks, modulated by task complexity, and implicate a potential disruption of whole-brain functional network hierarchy. In keeping with evidence of enhanced structural connectivity between the cognitive pre-SMA and motor cortex (149), these results may also provide a mechanistic explanation of cognition-triggered myoclonus in JME, i.e., *praxis induction* (141, 150). During the same working memory fMRI task, increased activation of the left dorso-lateral frontal cortex, on the other hand, was detected in JME patients with poorer decision-making performance (97). The latter may be interpreted as a compensatory mechanism to adequately engage working memory networks, required to carry out a complex decision-making task, and is reminiscent of findings in other neuropsychiatric disorders, such as schizophrenia (151, 152).

Other investigations in JME attempted to link the putative substrates of ictogenesis, likely represented by fronto-thalamo-cortical circuitry (150), with the associated cognitive comorbidities. O'Muircheartaigh and collaborators (129) demonstrated aberrant fronto-cortico-thalamic connectivity in JME during a verbal fluency fMRI task, which was associated with impoverished fluency performance. Complementary evidence was provided by a structural imaging analysis in recent-onset JME, which detected an association between performance on executive function tests and both thalamic and frontal volumes (140). On balance, this work suggests that the same circuitry accounting for seizure generation in JME may also mediate impairment of executive skills.

Other analyses sought to identify the neural correlates of cognitive traits in JME via structural imaging methods. Altered microstructural integrity of the supplementary motor area was associated with reduced performance on an expressive language task, while both gray matter volume and microstructural integrity of the posterior cingulate cortex related to mental flexibility (94). In a diffusion MRI tractography analysis, connectivity between post-central gyrus and precuneus was positively associated with verbal IQ, expressive language as well as verbal memory scores (143). Other studies, however, reported no correlations between white matter markers and a wide range of neuropsychological test scores, most of which relating to frontal lobe functions (95). While implicating midline frontal, primary sensory and parietal regions, structural imaging findings provide a less cohesive picture, as opposed to the more concordant evidence garnered via functional imaging studies.

Longitudinal investigations in new-onset JME may offer a window into the developmental trajectories of cognitive comorbidities. Lin et al. (142) documented lower response inhibition and psychomotor speed in patients with JME compared to controls at baseline, accompanied by persistence of intergroup differences after a 2 year follow-up, and more limited increase of general intelligence scores in the JME group. The latter cognitive traits were paralleled by structural abnormalities of high-order fronto-temporo-parietal association cortices, as demonstrated by an attenuation of the expected cortical thinning and contraction of surface areas. These findings overall implicate disrupted cortical maturation, and point to a post-migrational neurodevelopmental mechanism (142). Interestingly, further support to the neurodevelopmental hypothesis comes from recent analyses, indicating increased cortical folding complexity and inefficient cortico-cortical connectivity of orbitofrontal, ventrolateral frontal, premotor and temporo-polar areas. The latter regions also displayed abnormal cognitive network embedding, with fronto-parietal, dorsal attention and limbic cognitive systems being most affected (132).

Finally, a recent multi-modal imaging investigation in JME focused on the mesiotemporal lobe. Structural morphometric analyses indicated anomalies of hippocampal shape and positioning, pointing to altered mesiotemporal neurodevelopment during the prenatal stages, which related to reduced memory-related activation of both hippocampus and dorsolateral frontal areas (108). This work thus substantiates morphometric and functional abnormalities in JME extending beyond the classically involved fronto-cortico-thalamic or fronto-parietal systems, and supports functional relevance of mesiotemporal structural alterations, which reverberate on a fronto-temporal network subserving episodic memory.

### Neural Correlates of Cognitive Impairment in AE

As opposed to evidence in JME, direct assessments of the imaging correlates of cognitive function in AE are less numerous. Orbito-frontal and temporal lobe gray matter volumes were described as diminished in CAE (144), though formal correlations between the latter imaging measures and IQ scores were statistically significant in controls only. An investigation relating cortical thickness and sulcal depth to verbal and performance IQ found differential patterns of association between cognitive and structural measures in CAE compared to controls. Effects were particularly prominent for thickness and sulcal depth of medial/superior frontal and superior temporal areas, and implicated a negative relation between the latter and verbal IQ, which was instead positive in typically developing controls (114). In CAE, however, the authors identified positive associations between intelligence measures and thickness of the orbitofrontal cortex as well as sulcal depth of the middle frontal gyrus. Overall, these findings indicate distinct patterns of morphological signatures associated with general cognitive abilities, which may result from disease-related plasticity and reorganization.

Subsequent investigations assessed subcortical structures, in light of increasing evidence suggesting thalamic involvement in the generation of seizures and interictal discharges (137, 153, 154). While one study identified smaller thalamic volumes in CAE compared to controls, it did not detect a significant association between the latter and IQ measures (146). In JAE, reductions of gray matter volume and surface area were detected in the frontal, cingulate, and mesiotemporal locations, but formal correlations with cognitive measures were not available (155).

Functional imaging investigations in AE principally addressed the neural correlates of attention. During a sustained attention paradigm, an association was detected between lower activation of the medial frontal cortex and impaired task performance in CAE, which co-existed with reduced resting-state connectivity within an attentional network encompassing anterior insula and medial frontal cortex (145). More recently, combined behavioral and EEG-fMRI investigations detailed an association between (a) entity of functional activity changes within default-mode, fronto-parietal task-positive and sensorimotor-thalamic networks, and (b) intensity of absence seizures and related behavioral impairment. These findings thus provide direct evidence of a relationship between seizure-related cognitive compromise and levels of activity within large-scale brain networks (147).

## Determinants of Cognitive Dysfunction: Focus on Heritability

GGE are characterized by multi-factorial etiology and likely polygenetic underpinnings (156–158). A commonly held view regards GGE as heritable disorders of abnormal neurodevelopment, which may provide a unifying framework to understand vulnerability to seizure activity, distributed anomalies of functional and structural connectivity, as well as the associated cognitive and psychopathological comorbidities. Factors exerting additional modulation of the cognitive phenotype in GGE include disease-related variables, such as the combination of seizure types, seizure frequency and their responsiveness to treatment, disease duration, frequency of interictal epileptiform discharges, and specific effects of anti-epileptic medication (68, 107, 110).

Here, we will predominantly summarize research addressing genetic factors as determinants of cognitive impairment in GGE via family studies. Investigating neurobehavioral traits in first-order relatives of index cases provides the opportunity to account for potential effects of medication and seizures, whilst investigating individuals with comparable upbringing and socio-economic determinants. Common findings in patients and their relatives can be interpreted as intermediate phenotypes, or endophenotypes (159, 160) i.e., heritable traits co-segregating in affected families, underlying predisposition to disease and shedding light on its pathological mechanisms. Thus far, a few investigations have tested whether patterns of cognitive impairment in GGE may be heritable, and the majority of endophenotype research has focused on JME probands. While Levav et al. (121) detailed familial impairment in both JME and CAE samples, we are not aware of further subsyndrome-specific research in absence epilepsies or GGE-GTCS.

Levav et al. (121) demonstrated comparable deficits in attentional functioning for patients with GGE and their siblings relative to controls. More recently, Chowdhury et al. (71) showed that patients with GGE and first-degree relatives exhibited similar levels of impairment on tests of working memory, non-verbal reasoning, verbal fluency, and attention. In first-degree relatives, performances in the aforementioned domains mostly fell between patients and controls, suggesting a heritable component for cognitive impairment in GGE whilst implicating additional detrimental effects in patients, which may relate to a combination of seizures, anti-epileptic medication and/or greater genetic burden. In JME, two investigations described concomitant impairment of motor dexterity and phonemic fluency in probands and their siblings (92, 100). Semantic fluency and psychomotor speed also followed a similar trend, with relatives underperforming compared to controls. Interestingly, the familial similarities in cognitive performance were observed independent of abnormal interictal EEG in both studies. Furthermore, evidence suggests that JME probands and siblings both performed worse than controls during the memory formation and intention execution stages of a prospective memory task (51), which indicates heritability in relation to a complex cognitive skill, with tangible “everyday life” implications. Collectively, these investigations highlighted common neurobehavioural traits in patients and their unaffected siblings, mostly affecting executive function. Dyscognitive traits are thus implicated as a feature underpinned by genetic contribution, likely part of an extended disease-related phenotype, rather than mere consequence of seizure activity or anti-epileptic drug effects.

In parallel, recent imaging research complemented evidence on cognitive intermediate phenotypes. In patients with JME and their siblings, Wandschneider et al. (54) detected concomitant motor co-activation and abnormal connectivity between motor and prefrontal cognitive systems during a working-memory task, suggesting that altered interplay between functionally distinct macroscale networks may also be genetically driven. The previously detailed surface-based morphometry study, which investigated cortical folding complexity and cortico-cortical connectivity via a geodesic distance metric, identified concomitant abnormalities within high-order fronto-temporal cortices both in patients with JME and siblings. Similarly, abnormal embedding of the latter areas within large-scale cognitive networks, mostly affecting fronto-parietal, dorsal attention and limbic systems, was detected in both groups (132). Finally, recent work demonstrated co-segregation of abnormalities of hippocampal volume, shape and positioning both in patients with JME and their siblings, and showed their association with reorganization of both hippocampal and lateral frontal recruitment during a memory encoding functional MRI paradigm (108).

Collectively, these findings strongly indicate concomitant cognitive network abnormalities in patients with JME and their relatives, suggest involvement of cognitive domains beyond executive functions, and implicate high heritability.

## Conclusions

There is substantial evidence that GGE present with widespread cognitive impairment, predominantly involving executive functions. Cognitive profiles may slightly diverge across GGE subsyndromes, with absence epilepsies mostly affected in regard to phonological processing and attention, while high-level dysexecutive and risk-taking traits may be more prominent in JME. Studies assessing the neural correlates of cognitive dysfunction are more abundant in JME, and have frequently implicated thalamo-fronto-cortical and motor to prefrontal connections. In AE, on the other hand, there is evidence for a relationship between abnormal fronto-cortical morphometry and IQ, and impaired attention is paralleled by altered activation and connectivity within fronto-insular attentional networks. Whilst the etiology of cognitive impairment in GGE is likely multi-factorial, assessments of first-degree relatives, mostly of JME index patients, support heritability of cognitive profiles and the associated neural underpinnings, which qualify as suitable intermediate phenotypes (endophenotypes). Further research is awaited to (1) characterize profiles of cognitive impairment in homogeneous JAE samples, instead of assessing those along with CAE cases, irrespective of syndromic distinction; (2) elucidate patterns of dysfunction in GGE-GTCS; and (3) advance our insights into the pathological mechanisms of cognitive abnormalities, which may entail longitudinal investigation of cognitive trajectories in patients and their relatives, and, ultimately, require analyses of multi-source datasets encompassing neuropsychology, neuroimaging, genetics and neurophysiology.

## Author Contributions

CR, BW, MK, and LC planned the manuscript. CR and LC wrote the manuscript and carried out the subsequent revisions. CR and LC prepared the supporting material. PT and SB assisted in the interpretation of cognitive test results. All the co-authors provided substantial contributions to the first manuscript draft and subsequent revised versions.

### Conflict of Interest

The authors declare that the research was conducted in the absence of any commercial or financial relationships that could be construed as a potential conflict of interest.
